# Antiretroviral treatment regardless of CD4 count: the universal answer to a contextual question

**DOI:** 10.1186/s12981-016-0111-1

**Published:** 2016-07-26

**Authors:** Serge P. Eholié, Anani Badje, Gérard M. Kouame, Jean-Baptiste N’takpe, Raoul Moh, Christine Danel, Xavier Anglaret

**Affiliations:** 1 Service des Maladies Infectieuses et Tropicales, CHU de Treichville, Abidjan, Côte d’Ivoire; 2 Inserm U1219, Université de Bordeaux, Bordeaux, France; 3 Programme PACCI-ANRS Research Site, Abidjan, Côte d’Ivoire

**Keywords:** Early antiretroviral treatment, Randomized controlled trial

## Abstract

After a period where it was recommended to start antiretroviral therapy (ART) early, the CD4 threshold for treating asymptomatic adults dropped to 200/mm^3^ at the beginning of the 2000s. This was mostly due to a great prudence with regards to drug toxicity. The ART-start CD4 threshold in most international guidelines was then raised to 350/mm^3^ in 2006–2009 and to 500/mm^3^ in 2009–2013. Between 2012 and 2015, international guidelines went the last step further and recommended treating all HIV-infected adults regardless of their CD4 count. This ultimate step was justified by the results of three randomized controlled trials, HPTN 052, Temprano ANRS 12136 and START. These three trials assessed the benefits and risks of starting ART immediately upon inclusion (“early ART”) versus deferring ART until the current starting criteria were met (“deferred ART”). Taken together, they recruited 8427 HIV-infected adults in 37 countries. The primary outcome was severe morbidity, a composite outcome that included all-cause deaths, AIDS diseases, and non-AIDS cancers in the three trials. The trial results were mutually consistent and reinforcing. The overall risk of severe morbidity was significantly 44–57 % lower in patients randomized to early ART as compared to deferred ART. Early ART also decreased the risk of AIDS, tuberculosis, invasive bacterial diseases and Kaposi’s sarcoma considered separately. The incidence of severe morbidity was 3.2 and 3.5 times as high in HPTN052 and Temprano as in START, respectively. This difference is mostly due to the geographical context of morbidity. The evidence is now strong that initiating ART at high CD4 counts entails individual benefits worldwide, and that this is all the more true in low resource contexts where tuberculosis and other bacterial diseases are highly prevalent. These benefits in addition to population benefits consisting of preventing HIV transmission demonstrated in HPTN052, justify the recommendation that HIV-infected persons should initiate ART regardless of CD4 count. This recommendation faces many challenges, including the fact that switching from “treat at 500 CD4/mm^3^” to “treat everyone” not only requires more tests and more drugs, but also more people to support patients and help them remain in care.

## Background

### The optimal threshold for initiating ART: a 20-year quest

#### 1981–1996: The no-treatment era

During the first 15 years of the HIV pandemic, no treatment could sustainably control the replication of the virus and decrease mortality in HIV-infected people. The main direct and indirect consequence of the virus activity was infected and uninfected CD4 + T cells (CD4) death, causing progressively worsening immunosuppression, which exposed patients to the growing risk of opportunistic infection and death. The degree of immunosuppression and the subsequent risk of death over time were estimated with the CD4 count and with a clinical stage, as determined based on the opportunistic diseases that occurred in the patient. These two elements, CD4 count and clinical stage, enabled the setting of thresholds for some therapeutic decisions such as the initiation of prophylaxis for the major opportunistic infections. This notion of “threshold” is crucial: it influenced the administration of anti-HIV treatments for almost 30 years.

#### 1996: The sudden arrival of a highly potent treatment

In the mi-1990s, the arrival of combined antiretroviral therapy (ART) revolutionizes the disease prognosis [1, 2]. This era is characterised by a combination of three factors: the evidence that combination therapy has a much more potent and durable effect than monotherapy, the introduction of protease inhibitors, and the capacity to measure plasma viral load using the new molecular amplification techniques. By controlling viral replication, combination ART prevents cell death, increases CD4 count and restore immunity. This results in a dramatic decline in the risk of opportunistic infection and death [3]. In the first years of combination ART, “hit hard, hit early” becomes the mantra, with the hope that exerting maximal antiviral pressure on the virus during the initial phase of infection will prevent virus mutations and favour treatment success [4].

Combination ART controls the virus, however, it does not eradicate it. HIV reservoirs are shown to persist, even if plasma viremia is successfully suppressed for prolonged periods of time [5, 6]. From a fatal untreatable disease, HIV infection thus becomes a chronic disease requiring lifelong treatment. Like any chronic therapy, ART involves adherence issues and carries risks of cumulative toxicity; and like any anti-infective therapy, it carries risks of emergence of resistance, especially if it is not taken properly. To spare the patient unnecessary risks of toxicity and resistance, ART should therefore not be initiated “too early”—i.e. at an immuno-clinical stage where the treatment risks would outweigh the disease risks. This is where the quest for the “optimal immuno-clinical threshold for initiating ART” begins.

#### 1996–2014: Looking for the golden CD4 threshold

**Fig. 1:**
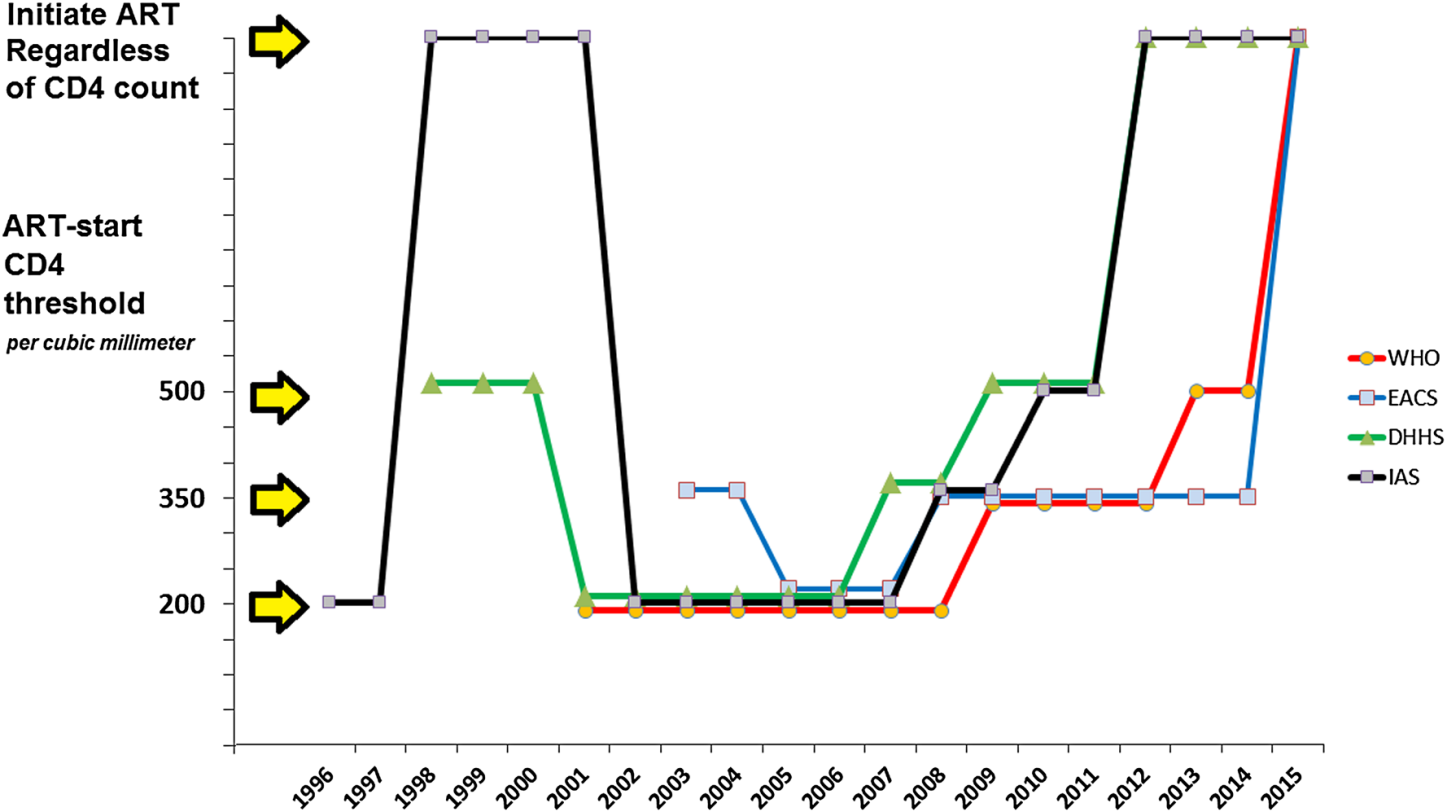
Temporal evolution of CD4 criteria to initiate ART in asymptomatic HIV+ adults (IAS, DHHS, EACS and WHO Guidelines)^(1)^. (1) Adapted from Marco Antonio Vitoria, WHO, Geneva. *cART* combined antiretroviral therapy. *DHHS* U.S. Department of Health and Human Services. *EACS* European AIDS Clinical Society. *IAS* International AIDS Society. *WHO* World Health Organisation

“When to start ART?” is essentially a risk/benefit question, but knowledge on benefits and risks changes over time. During the first 10 years of ART, the ART-start threshold fluctuates. After a short period in the late 1990s where it is recommended to start treatment from 500 CD4/mm^3^ without randomized trial evidence, threshold for treating asymptomatic adults drops to 200/mm^3^ at the beginning of the 2000s (Fig. 1) [7]. This shift is mostly due to a great prudence with regards to drug toxicity, especially cardiovascular or cerebrovascular, metabolic and renal. This is the time when, in order to spare toxicity, researchers carry out randomized trials of structured treatment interruption (STI), in which ART-treated patients whose CD4 count rises above 350/mm^3^ are proposed to temporarily interrupt their treatment [8, 9]. The underlying assumptions are that ART is the main determinant of all the non-HIV adverse effects and illness being observed in the early era of combination ART, and that ART’s primary goal is to maintain CD4 above 200/mm^3^. Therefore, any drug exposure beyond this target potentially entails unnecessary side effects.

In 2006, the results of the STI trials annihilate this approach. Contrary to the pre-trial hypotheses, the Trivacan and SMART trials show that patients who temporarily interrupt ART at the threshold of 350 CD4/mm^3^ have higher risk of severe morbidity compared to those who do not interrupt ART at the same threshold [8, 9]. In other words, below 350/mm^3^, HIV disease itself is more toxic than antiretroviral drugs. Furthermore, STI trial results no only suggest that people should worry more about starting ART earlier than about interrupting it. They shed important light on HIV morbidity, make us focus back on the virus, and illustrate the importance of the context.

In STI trials conducted in high resource countries, a significant part of the extra morbidity related to treatment interruptions is made up of non-infectious diseases—non-AIDS cancers, cardiovascular and renal diseases, and interruptions leads to a rise in inflammation markers [10, 11]. After more than 20 years of focusing on AIDS-defining opportunistic diseases (mostly infectious) at low CD4 counts, the focus thus becomes on non-AIDS HIV-related diseases (mostly non-infectious) at high CD4 counts. Part of these diseases being likely due to viral replication, rather than to immune suppression, the question is not whether the ART-start threshold will continue to rise anymore, but how high it will get.

In STI trials conducted in low resource countries, however, most of the extra morbidity related to treatment interruptions is made up of tuberculosis and invasive bacterial diseases [8, 12]. These infectious diseases are more frequent in poor settings than in rich settings. They are community diseases, but also act as opportunistic diseases, meaning that they are more frequent in HIV-infected individuals than in HIV-non infected ones. Thus, it is not that surprising that the overall risk of severe morbidity in HIV-infected patients at any CD4 count is higher in low resource settings as compared to high resource ones. Therefore, at the end of the 2000s, if the case for raising the ART-start threshold is already strong worldwide because of HIV non-AIDS non-infectious diseases, it is even stronger in regions where tuberculosis and bacterial diseases are more prevalent.

From there onwards the trend toward earlier initiation of ART is unstoppable. Two multicohort studies from industrialized countries suggest that 350/mm^3^ should be the minimum threshold for initiation of antiretroviral therapy [13, 14]. A randomized trial carried out in Haiti demonstrates that starting ART immediately in patients enrolled with less than 350 CD4/mm^3^ decreases the rates of death and incident tuberculosis compared to starting ART at 200 CD4/mm^3^ [15]. Between 2006 and 2009 the ART-start threshold is raised to 350/mm^3^ worldwide. Between 2009 and 2013, most guidelines further set the threshold to 500/mm^3^, the shift being quicker—with some exceptions—in high resource countries than in low resource ones. Raising the CD4 threshold to 500/mm^3^ is based on high-quality evidence from HPTN052 that ART reduces HIV transmission, but low-quality evidence from cohort studies that ART reduces mortality or progression to AIDS [16].

Finally, between 2012 and 2015, all international guidelines take the last step and recommend treating all HIV-infected adults regardless of their CD4 count [17–20]. This ultimate step is either corroborated or directly inspired by the results of three randomized controlled trials, HPTN 052, Temprano ANRS 12136 and START [21–24].

### HPTN 052, Temprano ANRS 12136 and START: the three early ART trials

**Fig. 2:**
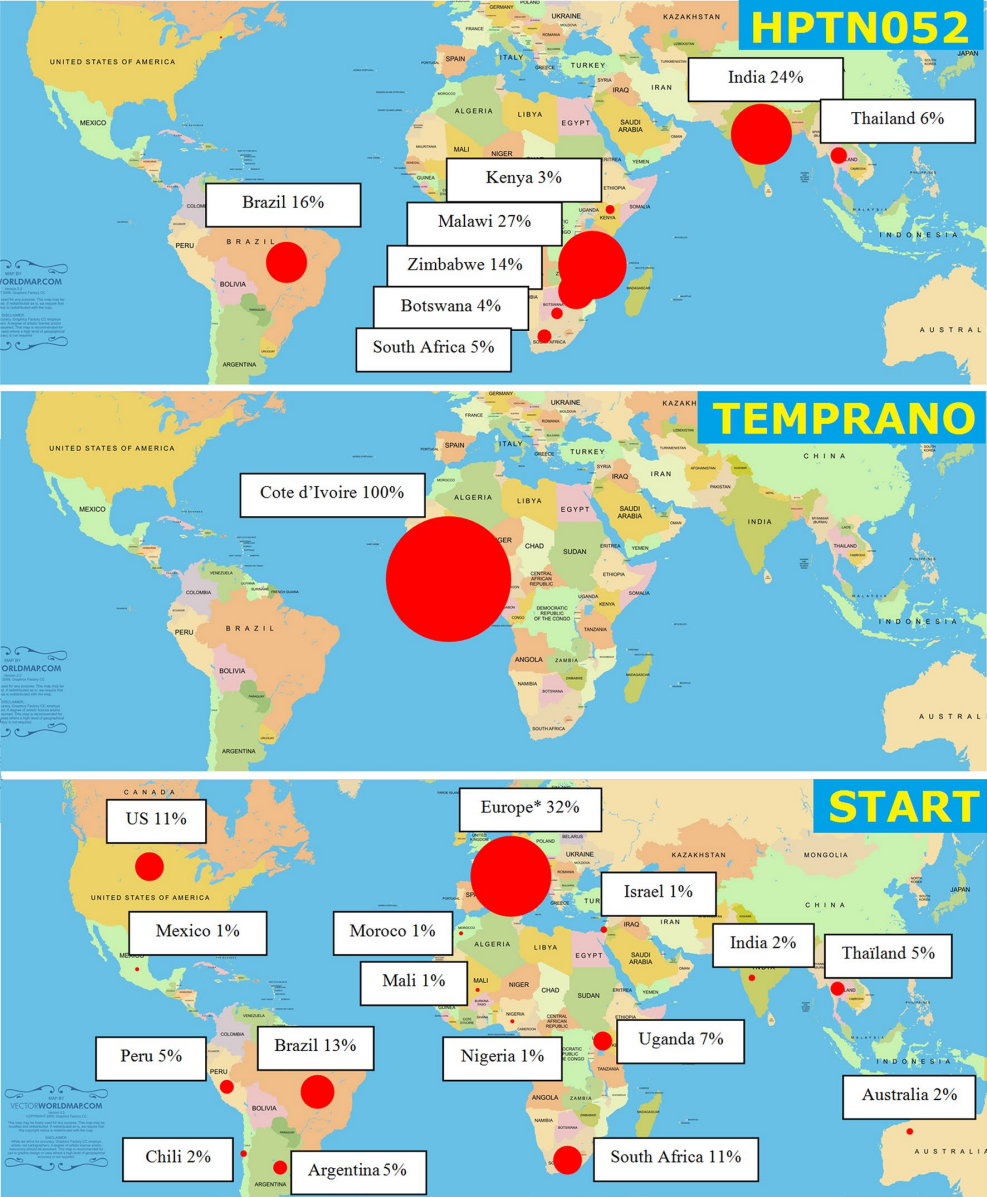
Geographical location of HPTN052, Temprano and START. *Source* Adapted from a Wikipedia map from http://www.vectorworldmap.com/; public domain. *Asterisks* recruitment for the START trial in Europe: Germany 7 %, United Kingdom 7 %, Spain 5 %, Belgium 2 %, France 2 %, Greece 2 %, Denmark 1 %, Italy 1 %, Poland 1 %, Portugal 1 %, Switzerland 1 %, others (Czech republic, Estonia, Finland, Luxemburg, Norway, Sweden, Ireland, all <1 %)

HPTN 052, Temprano and START were three open label randomized controlled trials. They took place between 2007 and 2015. Taken together, they recruited a total 8427 HIV-infected adults in 37 countries, including 48 % in Africa, 37 % in Europe, US, Australia or Israel, 17 % in Latin America, and 10 % in Asia (Table 1; Fig. 2).

**Table 1:** Main characteristics of the three early ART randomized controlled trials (HPTN052, Temprano ANRS 12136, START)

	HPTN052	Temprano	START
First enrolment–last visit	June 2007–May 2011	March 2008–Jan 2015	April 2009–May 2015
Protocol			
Study design	Open label RCT	Open label RCT	Open label RCT
CD4 inclusion criterion	350 < CD4 < 550	250/350 < CD4 < 800^b^	>500
ART initiation CD4 threshold^a^	250	200–350–500^b^	350
Composite primary outcome	Death, WHO stage 4, non-AIDS cancers, tuberculosis, severe bacterial infections, serious cardiovascular, diabetes mellitus	Death, AIDS, non AIDS cancers severe bacterial infections	Death, AIDS^c^, non AIDS cancer, serious cardiovascular^d^, renal failure, liver failure
Number of participants			
Enrolled/analyzed	1763/1761	2076/2056	4588/4585
Early ART	886	1033	2326
Deferred ART	875	1023	2359
Geographical origin			
Africa	54 %	100 %	21 %
Europe, USA, Australia, Israel	–	–	46 %
Asia	30 %	–	7 %
Latin America	16 %	–	25 %
Baseline characteristics			
% of women	49 %	79 %	27 %
Age, median (IQR)	33 (27–39)	35 (29–42)	36 (29–44)
CD4 count, median (IQR; Max)	436 (364–522; 550)	463 (366–572; 1456^e^)	651 (584–765; 2296)
Positive serum HBs Ag	5 %	9 %	NA
Follow-up characteristics			
Received IPT during trial follow-up, %	3 %	45 %	NA
Follow-up, year, median (IQR)	2.1 (1.5–2.9)	2.5 (2.5–2.5)	2.8 (2.1–3.9)

*RCT* randomized controlled trial; *IPT* isoniazid prophylaxis for tuberculosis

^a^ART-start CD4 threshold for asymptomatic patients randomized to the deferred ART strategy

^b^For ethical reasons, the Temprano investigators considered that the WHO revised guidelines had to be followed as soon as they were released

Therefore, they adopted the WHO 2010 guidelines in December 2009 as soon as the WHO rapid advice was released; and further adopted the WHO 2013 guidelines in August 2013. The 2010 and 2013 guidelines revisions impacted the trial procedures at two levels: the eligibility criterion ‘no WHO criteria for starting ART’, and the criteria for starting ART in participants assigned to the Deferred-ART strategy:

Asymptomatic patients were considered having ‘no WHO criteria for starting ART’ and therefore eligible for the trial: between the trial start and November 2009: if they had more than 250 CD4/mm^3^; Between December 2009 and July 2012: if they had more than 350 CD4/mm^3^; As enrolment was completed in July 2012, the WHO 2013 guidelines revision did not impact the eligibility criteria

Asymptomatic participants assigned to the Deferred-ART strategy started ART: between the trial start and November 2009: whenever they met the WHO 2006 criteria for starting ART (CD4 count <200/mm^3^); Between December 2009 and July 2013: whenever they met the WHO 2010 criteria for starting ART (CD4 count <350/mm^3^); Between August 1st 2013: whenever they met the WHO 2013 criteria for starting ART (CD4 count <500/mm^3^; or stable partnership with an HIV-negative individual)

^c^Excluding HSV and oesophageal candidiasis

^d^Myocardial infarction, stroke, or coronary revascularization

^e^Temprano, patients were eligible for the trial if the pre-inclusion CD4 count (measured within 1 months prior to randomization) was below 800/mm^3^. The “baseline” CD4 count distribution shown here is the distribution of values measured at inclusion (ie after informed consent), not the pre-inclusion value that determined eligibility. This explains why some patients had CD4 counts higher than 800/mm^3^ at baseline

The three trials had the same primary objective, that is to assess the benefits and risks of starting ART immediately upon inclusion (“early ART”) versus deferring ART initiation until the current institutional starting criteria were met (“deferred ART”). The primary outcome was severe morbidity, a composite outcome that included all-cause deaths, AIDS diseases, and non-AIDS cancers in the three trials. In addition to these three components, the primary outcome also included: severe bacterial diseases in Temprano and HPTN052; serious cardiovascular diseases in START and HPTN052; diabetes mellitus in HPTN052; and renal or liver failure in START. HPTN052 had a co-primary endpoint, HIV transmission to the partner, and was primarily powered to interrogate HIV transmission.

Asymptomatic patients were eligible for the trial if they had CD4 counts between 350 and 550/mm^3^ in HPTN052, between 250 and 800/mm^3^ in Temprano, and higher than 500/mm^3^ in START. As a consequence, the median CD4 count at baseline was 436/mm^3^ in HPTN052, 463/mm^3^ in Temprano and 651/mm^3^ in START, and the highest CD4 count at baseline was 550/mm^3^ in HPTN052, 1456/mm^3^ in Temprano and 2296/mm^3^ in START. In Temprano, 41 % of participants had more than 500 CD4/mm^3^ at baseline.

During the trial, asymptomatic patients randomized into the deferred ART strategy started ART at 250/mm^3^ in HPTN052 and 350/mm^3^ in START. In Temprano, the ART-start threshold was adjusted upward over time according to WHO guidelines updates, from 200/mm^3^ in 2008–2009 to 350/mm^3^ in 2009–2013 and 500/mm^3^ in 2013–2015. The median follow-up in the trials varied from 2.1 to 2.8 years.

The three trials had notable differences in terms of prevention and documentation of tuberculosis. Firstly, Temprano was a factorial 2 × 2 trial assessing in parallel two questions: early ART, and early INH preventive therapy (IPT). Therefore, participants were randomized not only to early or deferred ART, but also to start or not a 6-month course of IPT after 1 month of follow-up, provided they had no signs or symptoms evocative of tuberculosis. As a consequence, 45 % of participants in Temprano received IPT, versus only 3 % in HPTN052 and an unknown number in START. Secondly, in Temprano and HPTN052 all cases of confirmed and probable tuberculosis were documented and included in the primary outcome, while in START only confirmed cases with positive cultures were retained. The main results of the three trials were mutually consistent and reinforcing in terms of relative risks, while showing geographical context-specific absolute risks (Table 2).

**Table 2:** Main outcomes of the three early ART randomized controlled trials (HPTN052, Temprano ANRS 12136, START)

	Deferred ART		Early ART		Adjusted hazard ratio^f^ (95 % CI)
	N	Rate per 100 PY	N	Rate per 100 PY	
Composite primary outcome					
HPTN052	91	4.5	71	3.5	0.73 (0.52–1.03)
Temprano	111	4.9	64	2.8	0.56 (0.41–0.76)
Baseline CD4 < 500	73	5.5	41	3.0	0.56 (0.38–0.83)
Baseline CD4 ≥ 500	38	4.1	23	2.4	0.56 (0.33–0.94)
START	96	1.4	42	0.6	0.43 (0.30–0.62)
Separated outcome					
Death^a^					
HPTN052	15	NA	11	NA	0.73 (0.34–1.59)
Temprano	26	1.9	21	0.8	0.80 (0.45–1.40)
START	21	0.3	12	0.2	0.58 (0.28–1.17)
AIDS^a^					
HPTN052	61	NA	40	NA	0.64 (0.43–0.96)
Temprano	65	2.8	33	1.4	0.50 (0.33–0.76)
START	50	0.7	14	0.2	0.28 (0.15–0.50)
Tuberculosis^a,b^					
HPTN052	34	1.8	17	0.8	0.49 (0.28–0.89)
Temprano	55	2.4	28	1.2	0.50 (0.32–0.79)
START	20	0.3	6	0.1	0.29 (0.12–0.73)
AIDS and non-AIDS malignancies^a,c^					
HPTN052	7	NA	4	NA	NA
Temprano	6	NA	3	NA	NA
START	39	NA	14	NA	NA
Invasive bacterial diseases^d^					
HPTN052	13	NA	20	NA	NA
Temprano	36	1.5	14	0.6	0.39 (0.21–0.71)
START	36	0.5	14	0.2	0.38 (0.20–0.70)
Serious cardiovascular^e^					
HPTN052	3	NA	1	NA	NA
Temprano	6	NA	3	NA	NA
START	14	0.20	12	0.17	0.84 (0.39–1.81)

*N* number of participants who had at least one such type of outcome; *ART* antiretroviral treatment; *NA* non available; *PY* person-years

^a^Component of the composite primary outcome in the three trials

^b^Total number of pulmonary and extra pulmonary TB episodes recorded in the three trials: HPTN052: pulmonary, n = 30 (early: 14; deferred: 16); extra-pulmonary, n = 20 (early: 3; deferred: 17); Temprano ANRS 12136: pulmonary, n = 43 (early: 19; deferred: 24); extra-pulmonary, n = 41 (early: 9; deferred: 33). START: pulmonary, n = 23 (early: 6; deferred: 17); extra-pulmonary, n = 3 (early: 0; deferred: 3)

^c^Cervical carcinoma, Kaposi’s sarcoma, Lymphoma, Hodgkin’s, Lymphoma, non-Hodgkin’s non-AIDS cancers

^d^Invasive bacterial diseases were a component of the composite primary outcome in Temprano and HPTN052, and a secondary outcome in START

^e^Serious cardiovascular diseases were a component of the composite primary outcome in START and HPTN052, and a secondary outcome in Temprano

^f^The trials analyses were adjusted for geographic regions (START) or study site (HPTN 052 and Temprano). In Temprano, Hazard Ratios were also adjusted for the IPT/no IPT treatment

In terms of absolute risks, the incidence of severe morbidity in the deferred ART strategy was 3.2 and 3.5 as high in HPTN052 and Temprano compared to START, respectively. This difference is likely explained by the context (more patients in low resource settings in HPTN052 and Temprano), the population (higher baseline CD4 count in SMART), and the differences in the documentation of tuberculosis discussed above.

In terms of relative risks, the overall risk of severe morbidity was 44–57 % lower in patients randomized to the early ART strategy as compared to those randomized to the deferred ART strategy in Temprano and START, respectively. In Temprano, the reduction in severe morbidity was of similar magnitude and remained significant when restricting the analysis to patients who had more than 500 CD4/mm^3^ at baseline. In HPTN052, the overall difference did not reach significance.

When considering separately each component of the composite primary outcome or important secondary outcomes, early ART significantly decreased the risk of: AIDS in the three trials; tuberculosis considered separately in the three trials; invasive bacterial diseases in Temprano and SMART; and Kaposi’s sarcoma in SMART. These are secondary analysis whose power may be limited by the number of events in each group. As shown in Table 2, the number of AIDS or non-AIDS HIV-related events was lower with early ART than in deferred ART in almost every subgroup, even if this difference did not always reach significance. Of note, in Temprano, the trial who assessed in parallel the efficacy of early ART and IPT, IPT also reduced the risk tuberculosis by 66 % overall, 68 % in patients who had less than 500 CD4/mm^3^ at baseline and 63 % in those who had more than 500 CD4/mm^3^ at baseline. The efficacy of ART and IPT in reducing tuberculosis was independent [22].

Finally, in the three trials, ART appeared to be equally well tolerated in both strategies (data not shown).

## Discussion

These three trials were conducted in different geographical contexts and in populations at various immunosuppression stages. This diversity strengthens their common conclusion: all over the world, HIV-infected persons should be recommended to initiate ART regardless of CD4 count. ART initiation should not be triggered by any clinical or immunological threshold anymore, and the objective of ART clearly becomes to suppress viral replication and prevent—rather than cure—inflammation and immune deficiency.

Broadening treatment eligibility directly leads to increase the number of individuals treated. Switching from treating at 500 CD4/mm^3^ to treating all HIV-infected persons irrespective of CD4 count meets many barriers and challenges, especially in low resource settings. Of the 35 million people living with HIV, about 19 million do not even know that they are HIV-positive [25]. The majority of those who know their status still present to care late with low CD4 counts [26]. We need a robust testing agenda, including innovative approaches such as community-based testing and self-testing to encourage early testing [27]. Furthermore, testing and initiating treatment early is not worth spending energy on it if patients stop treatment afterwards. Therefore, initiating ART irrespective of CD4 count in settings with high HIV burden requires increased capacity not only in terms of tests and drugs, but also in terms of infrastructures and trained staff empowered to support patients [28]. In resource-constrained settings, a phased approach to implementation may be needed [29, 30].

Finally, the randomized trials have shown that the benefits/risk ratio of starting ART irrespective of CD4 count is favorable. However, if ART at any CD4 count is proven to have strong benefits globally, the question remains open of whether these benefits may outweigh the risks in subgroups of patients, such as HIV controllers [31]. Therefore, now that we have given a global answer to the question of ‘when to start ART’, we should explore to question of ‘when not to start ART immediately’ at the individual level [16]. Should these challenges be addressed, recommending initiating ART irrespective of CD4 count may bring benefits that extend beyond the individual benefits demonstrated in HPTN 052, Temprano and START. A person who starts treatment earlier is less exposed to the initial complications of the treatment such as immune constitution inflammatory syndrome (IRIS), and thus easier to manage. In addition, not having to wait for a CD4 count result to decide whether to initiate ART should facilitate and shorten the pre-treatment period. These two advantages should accelerate task delegation and help decentralize care, thus increasing the likelihood that the UNAIDS target of 90 % of all people with diagnosed HIV infection receiving sustained ART will be reached by 2020 [32] . In addition, ART does not just reduce morbidity in the treated individuals, it also reduces the number of people with a detectable viral load. It thus contributes to reducing HIV transmission and to controlling the HIV epidemic [24]. Finally, in the many countries where HIV and tuberculosis are both highly prevalent, ART and IPT independently decrease the risk of tuberculosis in HIV-infected patients, even at high CD4 counts. Therefore, initiating ART early should help decrease the risk of tuberculosis transmission and further contribute to tuberculosis control [33] and IPT should be given even to patients who start ART at high CD4 counts [22].

## Conclusions

In conclusion, the evidence is now strong that initiating ART at high CD4 counts entails individual benefits, and that this is all the more true for those who live in low resource contexts. These individual benefits in addition to previously demonstrated population benefits, consisting of preventing HIV transmission to non-HIV-infected partners, fully justify the universal recommendation that HIV-infected persons should be recommended to initiate ART regardless of CD4 count. This recommendation faces many challenges, including the fact that switching from “treat at 500 CD4/mm^3^” to “treat everyone” is not only a matter of more tests and more drugs. It also requires more people to support patients and help them remain in care once they started treatment. Over the past years, the “Test and Treat” model has evolved to “Seek, Test, Treat and Retain”. Until HIV can be eradicated, it should now be formulated this way: “Seek, test and treat every HIV infected persons, and empower staff and patients to ensure lifelong treatment continuity” [28].
